# Feasibility, safety, and preliminary clinical efficacy of resting-state functional connectivity MRI-guided rTMS for adolescents with major depressive disorder: Protocol for a randomized, double-blind, three-arm, sham-controlled pilot trial

**DOI:** 10.1371/journal.pone.0355676

**Published:** 2026-08-06

**Authors:** Hongyi Chen, Danyu Xu, Wenjing Wang, Xinyin Luo, Xiaocong Jiang, Ningyi Wan, Yuhuan Wei, Ting Wang, Li Zhang, Xuemei Li, Xinyu Zhou

**Affiliations:** 1 Department of Psychiatry, Key Laboratory of Major Brain Disease and Aging Research (Ministry of Education), the First Affiliated Hospital of Chongqing Medical University, Chongqing, China; 2 Department of Psychology, the Second Affiliated Hospital of Chongqing Medical University, Chongqing, China; Public Library of Science, UNITED STATES OF AMERICA

## Abstract

**Background:**

Major depressive disorder (MDD) is highly prevalent in adolescents, and a substantial proportion of patients show inadequate response or limited tolerability to first-line treatments such as selective serotonin reuptake inhibitors and cognitive behavioral therapy, highlighting the need for additional treatment options. Repetitive transcranial magnetic stimulation (rTMS) has demonstrated efficacy in adults with depression, but evidence in adolescents remains limited. Resting-state functional connectivity MRI (rs-fcMRI) may provide a circuit-informed approach for individualizing rTMS target selection within the dorsolateral prefrontal cortex (DLPFC) based on its functional connectivity with the subgenual anterior cingulate cortex (sgACC).

**Methods:**

This is a randomized, double-blind, three-arm, sham-controlled pilot trial. Forty-five adolescents with MDD will be assigned in a 1:1:1 ratio to conventional left DLPFC-targeted rTMS, rs-fcMRI-guided rTMS targeting the individualized DLPFC site showing the strongest negative connectivity with the sgACC, or sham stimulation. Treatment will be delivered over 4 weeks. Primary outcomes will include recruitment feasibility, intervention adherence, 6-month retention, preliminary clinical efficacy, and safety, assessed by enrollment, completion of 20 treatment sessions, completion of 6-month follow-up, CDRS-R-based response and remission, and adverse events/serious adverse events. Secondary outcomes will include depressive and anxiety symptoms, suicidality, sleep quality, global clinical status and improvement, rumination, and pediatric health-related quality of life.

**Discussion:**

This pilot trial will provide preliminary evidence regarding the feasibility, acceptability, safety, and early clinical efficacy signals of rs-fcMRI-guided rTMS in adolescents with MDD. By comparing individualized circuit-informed targeting with conventional left DLPFC stimulation and sham stimulation, the study may help clarify whether this imaging-guided neuromodulation approach is practicable and clinically informative in this population. The findings are expected to support refinement of study procedures, outcome selection, and trial design for a future fully powered randomized controlled trial.

**Trial registration:** ClinicalTrials.gov NCT07185438.

## Introduction

### Burden of adolescent MDD and persistent treatment challenges

Adolescence is a sensitive developmental period characterized by rapid neurobiological maturation and the consolidation of socioemotional functioning. During this stage, the ongoing development of cognitive control and emotion regulation systems, together with family, academic, and interpersonal stressors, increases vulnerability to internalizing psychopathology. Major depressive disorder (MDD) is among the most prevalent psychiatric disorders in adolescents and is associated with substantial impairment in physical health, educational attainment, and long-term psychosocial functioning.

According to the World Health Organization (WHO), approximately one in seven adolescents aged 10–19 years lives with a mental health condition, and depression is among the leading causes of illness and disability in this age group [1]. Core clinical features of adolescent MDD include persistent low mood, anhedonia, cognitive dysfunction, and reduced self-esteem; in severe cases, suicidal ideation and suicidal behaviors may occur. In China, national surveillance data indicate that suicide is a leading cause of death among individuals aged 15–24 years, with depression representing a major underlying psychological risk factor [2]. Given its high prevalence, early onset, and potentially enduring consequences, adolescent MDD remains a major public health concern.

Current clinical guidelines recommend selective serotonin reuptake inhibitors (SSRIs), often in combination with cognitive behavioral therapy (CBT), as first-line treatment for adolescent depression [3,4]. However, real-world effectiveness remains suboptimal, and treatment response is often incomplete or unstable. Approximately 30%–40% of adolescents show inadequate response to conventional pharmacotherapy and remain at elevated risk of relapse [5,6]. In addition, SSRI treatment in adolescents is frequently accompanied by concerns regarding agitation, possible worsening of suicidal ideation, weight changes, and sexual dysfunction, all of which may compromise adherence and limit sustained benefit [7,8]. Collectively, these limitations highlight the need for safe, non-pharmacological, and more precisely targeted interventions for adolescent MDD.

### rTMS for MDD: Rationale and evidence gaps in adolescents

Repetitive transcranial magnetic stimulation (rTMS) is a non-invasive neuromodulation technique that uses rapidly changing magnetic fields to induce electrical currents in cortical tissue, thereby modulating cortical excitability and distributed neural circuits involved in emotional regulation, cognitive processing, and behavioral control [9]. Owing to its favorable safety profile and demonstrated antidepressant effects, rTMS received U.S. Food and Drug Administration (FDA) clearance in 2008 for treatment-resistant depression in adults [10]. Extensive adult studies have shown that high-frequency stimulation of the left dorsolateral prefrontal cortex (DLPFC) can significantly reduce depressive symptom severity, with adverse effects generally being mild and transient, most commonly scalp discomfort and headache [11,12].

Over the past decade, interest in applying rTMS to adolescent MDD has increased. Nevertheless, the current evidence base remains insufficient to support definitive conclusions regarding its efficacy in this population. Preliminary studies suggest that rTMS is generally well tolerated and may offer therapeutic benefit; however, much of the existing literature is limited by methodological shortcomings, including open-label designs, small sample sizes, short follow-up periods, and a lack of adequately powered randomized controlled trials (RCTs) [13,14]. Notably, the multicenter, double-blind, sham-controlled RCT by Croarkin et al. (2021) did not demonstrate a significant between-group difference in Hamilton Depression Rating Scale improvement [15]. Additional limitations across existing studies include predominantly single-center recruitment, limited generalizability, insufficient long-term follow-up to evaluate durability, reliance on conventional left DLPFC targeting without accounting for inter-individual neuroanatomical and network-level variability, and the absence of biomarkers to guide treatment selection or predict response.

Taken together, although rTMS is a promising intervention for adolescent MDD, important questions remain regarding its feasibility, acceptability, safety, and preliminary clinical efficacy in youth, particularly when more individualized and biologically informed targeting strategies are used.

### Circuit-informed targeting: DLPFC-sgACC functional connectivity as a precision strategy

Accumulating evidence suggests substantial inter-individual variability in DLPFC functional connectivity, which may contribute to heterogeneity in clinical response to standard DLPFC stimulation. The functional connectivity targeting framework proposed by Fox and colleagues posits that the most effective rTMS targets within the DLPFC are those showing the strongest negative functional connectivity with the subgenual anterior cingulate cortex (sgACC), a key node implicated in depression-related network dysfunction [16,17]. This framework has prompted growing interest in individualized target localization using functional magnetic resonance imaging (fMRI), particularly resting-state functional connectivity MRI (rs-fcMRI).

In adults, several studies suggest that fMRI-guided rTMS may outperform conventional targeting approaches in both symptom improvement and circuit-level modulation [18]. For example, Drysdale et al. (2017) and Cash et al. (2021) reported that stimulation of the left DLPFC subregion showing the strongest negative functional connectivity with the sgACC was associated with improved response rates and partial normalization of dysfunctional connectivity patterns [19,20]. However, MRI-guided rTMS RCTs in adolescents with MDD remain lacking. This gap is clinically important because adolescence is characterized by heightened neuroplasticity and ongoing maturation of fronto-limbic circuitry, suggesting that circuit-informed neuromodulation may have developmentally specific effects in this age group. Supporting this view, Seewoo et al. (2022) reported neurostructural changes following rTMS in adolescents that differed from those observed in adults, underscoring the need for more refined and individualized treatment strategies in youth [21].

Accordingly, inter-individual differences in DLPFC-sgACC connectivity may help explain why some adolescents do not respond to conventional rTMS targeting, and rs-fcMRI-guided neuromodulation may offer a more precise therapeutic approach during this sensitive developmental window.

### Significance and novelty

Against this background, we propose a randomized, double-blind, three-arm, sham-controlled pilot trial to evaluate the feasibility, acceptability, safety, and preliminary clinical efficacy of rs-fcMRI-guided rTMS for adolescents with MDD. Using an intelligent neuronavigation system, individualized stimulation targets will be generated by identifying, for each participant, the DLPFC location showing the strongest negative functional connectivity with the sgACC on rs-fcMRI. This design will allow direct comparison among conventional left DLPFC-targeted rTMS, rs-fcMRI-guided individualized rTMS, and sham stimulation.

The primary outcomes of this pilot trial include feasibility, safety, and preliminary clinical efficacy. Feasibility will be assessed through recruitment feasibility, intervention adherence, and retention rate at the 6-month follow-up. Safety will be evaluated by monitoring the incidence of adverse events and serious adverse events throughout the treatment and follow-up periods. Preliminary clinical efficacy will be assessed by depressive symptom response and remission based on the Children’s Depression Rating Scale-Revised (CDRS-R), with response defined as a reduction of at least 50% in the CDRS-R total score from baseline and remission defined as a CDRS-R total score of 28 or lower. In addition, this study will explore whether aberrant connectivity patterns within depression-relevant circuits show evidence of modulation following targeted neuromodulation.

Overall, this pilot trial is expected to provide early evidence regarding the practicality and safety of rs-fcMRI-guided rTMS in adolescent MDD, while generating preliminary clinical and neuroimaging signals to refine study procedures and inform the design and outcome selection of a future fully powered randomized controlled trial. In doing so, it may help advance the development of more individualized neuromodulation strategies for adolescent depression.

## Methods

### Study design and setting

This investigator-initiated, single-center pilot trial is designed to evaluate the feasibility, safety and preliminary clinical efficacy of rs-fcMRI-guided rTMS in adolescents with MDD. Participants will be randomly assigned to one of three parallel arms: conventional left dorsolateral prefrontal cortex (DLPFC)-targeted rTMS, rs-fcMRI-guided rTMS targeting an individualized DLPFC site defined by DLPFC-sgACC functional connectivity, or sham rTMS.

The study adopts a randomized, double-blind, three-arm, sham-controlled, parallel-group pilot design and will be conducted at the First Affiliated Hospital of Chongqing Medical University (Chongqing, China) from September 2025 to September 2027. A total of 45 adolescents will be enrolled, with 15 participants allocated to each group in a 1:1:1 ratio. Participant recruitment commenced in September 2025 and is expected to be completed by December 2026. Recruitment will be monitored throughout the study to assess enrolment feasibility and ensure timely participant accrual. At the time of manuscript submission, the study is ongoing, and no study results have been generated. Data collection is expected to be completed by June 2027, and study results are anticipated to be available by September 2027.

The study protocol was approved by the Ethics Committee of the First Affiliated Hospital of Chongqing Medical University (Ethical Approval No. 2025-533-01) and registered at ClinicalTrials.gov (NCT07185438).

This pilot trial consists of a 7-day screening period, a 4-week intervention phase, and follow-up assessments up to 6 months. After eligibility has been confirmed, participants and their legal guardians will provide written informed consent, and participants will undergo baseline assessments, including MRI. Participants will then be randomly assigned to one of three groups to receive a 4-week course of active or sham rTMS. Follow-up assessments will be performed at weeks 8, 16, and 24 to evaluate clinical outcomes and safety over time. Cognitive and multimodal measures, including EEG, fNIRS, eye-tracking measures, facial expression features, and acoustic features, will also be collected at baseline, at the end of treatment, and during follow-up as exploratory measures.

**Fig 1:**
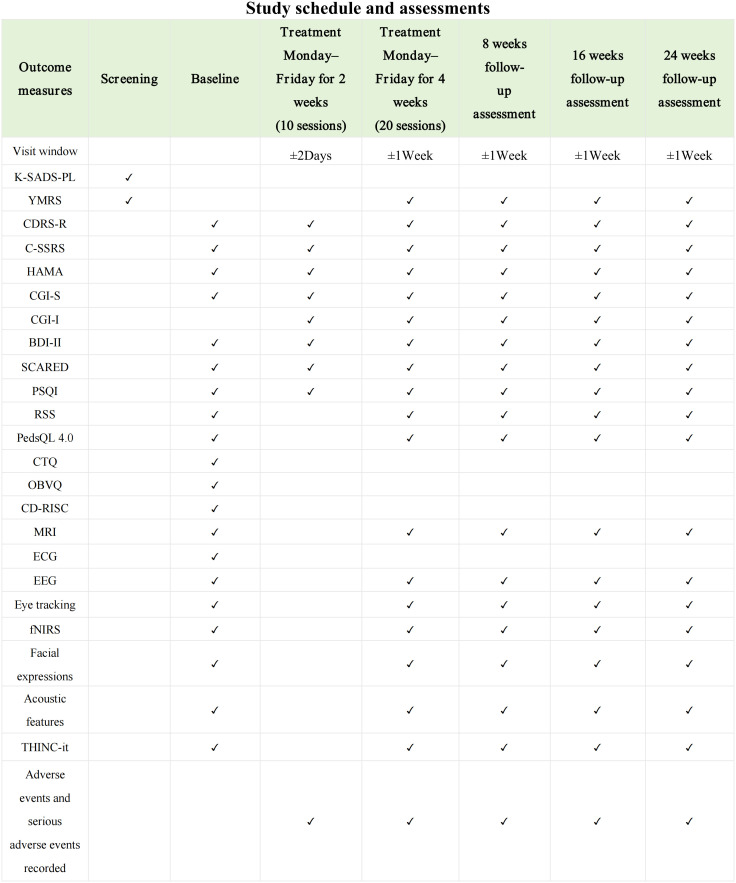
Study schedule and assessments.

**Fig 2:**
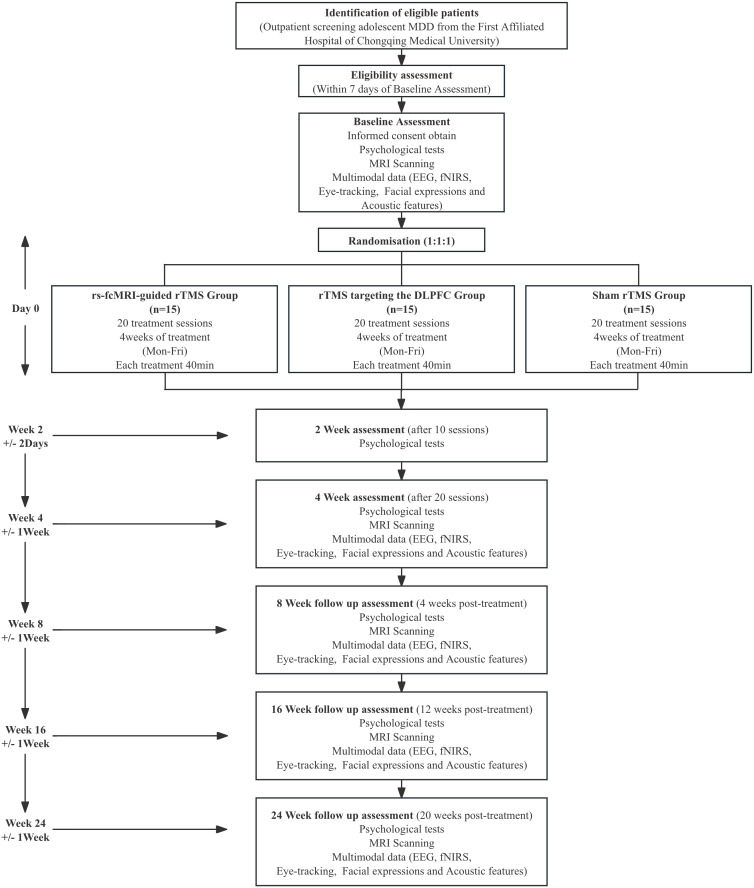
Study flow chart.

In addition, study flow will be documented by recording the numbers of participants who are screened, excluded, randomized, treated, lost to follow-up, or withdrawn, together with reasons for non-participation, exclusion, or discontinuation. The schedule of enrolment, intervention, and assessments is presented in Fig 1 (SPIRIT schedule), and the overall study flow is shown in Fig 2 (Study flow chart).

Schedule of study. K-SADS-PL, Kiddie Schedule for Affective Disorders and Schizophrenia-Present and Lifetime version; YMRS, Young Mania Rating Scale; CDRS-R, Children’s Depression Rating Scale-Revised; C-SSRS, Columbia-Suicide Severity Rating Scale; HAMA, Hamilton Anxiety Rating Scale; CGI-S, Clinical Global Impression-Severity Scale; CGI-I, Clinical Global Impression-Improvement Scale; BDI-II, Beck Depression Inventory-II; SCARED, Screen for Child Anxiety Related Emotional Disorders; PSQI, Pittsburgh Sleep Quality Index; RRS, Rumination on Responses Scale; PedsQL 4.0, Pediatric Quality of Life Inventory 4.0 Generic Core Scales; OBVQ, Olweus Bullying Victimization Questionnaire; CD-RISC, Connor-Davidson Resilience Scale; ECG, Electrocardiogram; EEG, electroencephalographic; fNIRS, functional Near-Infrared Spectroscopy.

Flow diagram for the trial. MDD, major depressive disorder; EEG, electroencephalographic; fNIRS, functional Near-Infrared Spectroscopy; DLPFC, dorsolateral prefrontal cortex; sgACC, subgenual anterior cingulate cortex; rTMS, repetitive transcranial magnetic stimulation.

### Participants

Researchers will perform eligibility screening based on inclusion and exclusion criteria. All participants must provide voluntary informed consent and sign the informed consent form after confirming their eligibility.

### Inclusion criteria

Participants who meet the following criteria are eligible to participate in this study.

1. Age 12**–**18;
2. Diagnosis of MDD according to the Diagnostic and Statistical Manual of Mental Disorders, Fifth Edition (DSM-5), confirmed through the Kiddie Schedule for Affective Disorders and Schizophrenia-Present and Lifetime version (K-SADS-PL), currently in a depressive episode [22,23];
3. Score ≥ 40 on Children’s Depression Rating Scale-Revised (CDRS-R);
4. Stable pharmacological treatment: At least 4 weeks of stable psychiatric medication use prior to enrollment, with continuation of the same psychiatric medication regimen throughout the study.

### Exclusion criteria

Participants meeting any of the following criteria will be excluded from this study.

1. Psychiatric comorbidities other than anxiety disorders;
2. Depression with psychotic symptoms;
3. Young Mania Rating Scale (YMRS) score >13;
4. A history of neurological disorders (e.g., epilepsy, brain injury) or severe somatic diseases (e.g., thyroid disorders, lupus, diabetes, pulmonary, hepatic, or renal impairment, major trauma);
5. Patients currently using anticonvulsants or high-dose benzodiazepines;
6. A history of electroconvulsive therapy (ECT), transcranial magnetic stimulation (TMS), transcranial direct current stimulation (tDCS), transcranial alternating current stimulation (tACS), or other neuromodulation treatments;
7. A history of alcohol or substance abuse or dependence;
8. Women who are pregnant or breastfeeding;
9. Current high suicide risk;
10. Potential complicating factors related to transcranial magnetic stimulation, such as scalp conditions or perforations that may affect magnetic field delivery;
11. Contraindications to MRI.

### Sample size

As a pilot trial, this study is not designed or powered to test definitive treatment efficacy. A total of 45 MDD adolescents will be enrolled, with 15 participants allocated to each of the three study arms. The sample size was determined pragmatically on the basis of feasibility, anticipated clinical capacity, and available study resources. This sample size is intended to provide estimates of recruitment feasibility, intervention adherence, retention, safety, and preliminary clinical efficacy signals, as well as variability parameters needed to refine the protocol and inform the sample size calculation for a future fully powered randomized controlled trial.

### Randomization

Randomisation will be conducted before the first treatment session [24]. Participants will be assigned in a 1:1:1 ratio to the rs-fcMRI-guided rTMS group, the conventional left DLPFC-targeted rTMS group, or the sham rTMS group [25,26]. The allocation sequence will be generated by an independent statistician who is not involved in participant recruitment, intervention delivery, or outcome assessment, using a computer-generated permuted block randomisation scheme with a block size of 9.

Allocation concealment will be ensured using sequentially numbered, sealed, opaque envelopes, which will be opened immediately before the first treatment session. The assigned study number will determine treatment allocation throughout the trial. rTMS will be delivered by certified operators who, because of the nature of the intervention, will not be blinded to treatment condition; however, they will not be involved in recruitment, clinical evaluation, or outcome assessment.

### Blinding

Participants, treating clinicians responsible for routine clinical care, and outcome assessors will remain blinded to treatment allocation throughout the trial [27]. Any instance of inadvertent unblinding of an outcome assessor will be documented in detail, and all subsequent assessments for that participant will be conducted by an alternative blinded assessor. After completion of the intervention, outcome assessors will be asked to guess participants’ group assignment in order to evaluate the integrity of blinding.

Personnel involved in treatment delivery, including unblinded staff and certified rTMS operators, will be strictly excluded from recruitment, clinical evaluation, outcome assessment, and data analysis to minimize the risk of bias.

### Interventions

The primary intervention in this trial is rTMS. To minimize pharmacological confounding, participants must have been on a stable psychiatric medication regimen for at least 4 weeks before enrolment and are required to maintain the same regimen throughout the stimulation phase and follow-up period, unless treatment modification is clinically indicated.

The sham coil is designed to closely mimic the appearance and sensory experience of active rTMS, thereby helping to maintain blinding among participants. Any necessary adjustments to stimulation intensity will be made on the basis of tolerability and safety considerations and will be closely monitored throughout treatment.

All 20 treatment sessions will be delivered in the Department of Psychiatry at the First Affiliated Hospital of Chongqing Medical University by experienced physical therapists trained in standardized rTMS procedures.

### rTMS delivery and general procedures

rTMS will be delivered using the Black Dolphin TMS robotic system (Spirit Dolphin, SLD-YXRJ-V1.0, Solide Company, Xi’an, China) equipped with a figure-of-eight coil. Individualized neuronavigation will be supported by a personalized three-dimensional facial tracking model generated from each participant’s MRI data, allowing the robotic arm to position the coil accurately over the predefined stimulation target across treatment sessions.

Before the first treatment session, the resting motor threshold (RMT) will be determined for each participant. The RMT is defined as the minimum stimulation intensity required to elicit motor-evoked potentials in at least 5 of 10 trials. Participants in all three study arms will receive a 4-week course of active or sham stimulation consisting of 20 weekday sessions, with identical treatment frequency and procedural workflow across groups.

### Stimulation targets

Participants in the rs-fcMRI-guided rTMS group will receive stimulation at an individualized left DLPFC target identified under MRI guidance as the voxel showing the strongest negative resting-state functional connectivity with the sgACC. Participants in the conventional left DLPFC-targeted rTMS group will receive MRI-guided stimulation at the predefined left DLPFC target. Participants in the sham rTMS group will receive sham stimulation matched in appearance, sound, and procedural experience to active treatment in order to help maintain participant blinding.

### Stimulation parameters

For both active treatment groups, stimulation parameters will be identical: frequency 10 Hz, intensity 120% of the individual RMT (reducible to 110% of RMT during week 1 if not tolerated), 2400 pulses per session, and a session duration of approximately 40 minutes. Treatment will be administered once daily on weekdays for a total of 20 sessions over 4 weeks.

In the sham group, participants will undergo the same treatment schedule and procedural workflow as those in the active groups, but stimulation will be delivered with a sham coil that reproduces the acoustic and tactile sensations of active rTMS while minimizing meaningful magnetic field penetration.

### Concomitant medication management

Participants must be receiving a stable pharmacological treatment regimen, defined as no changes for at least 4 weeks before enrolment, and are required to maintain this regimen throughout the trial unless modification is clinically indicated. Concomitant medications and any treatment adjustments will be systematically documented at each study visit to minimize potential confounding arising from medication changes.

For analyses of preliminary clinical efficacy, baseline medication-related factors will be considered as covariates in the primary outcome model to support estimation of treatment effects under relatively stable pharmacological conditions. Pre-specified sensitivity analyses will also be conducted to examine the robustness of the findings under alternative medication scenarios and protocol deviations. Collectively, these procedures are intended to facilitate interpretation of treatment effects within a relatively stable medication context.

### Discontinuation of the intervention

Participants may withdraw from the study at any time without penalty or any effect on their routine clinical care. Data collected before withdrawal may be retained for analysis unless the participant requests otherwise, and no further study procedures or data collection will be performed after withdrawal. Where feasible, the study team will attempt to contact participants who withdraw in order to document the reasons for discontinuation.

Participants will discontinue the intervention or be withdrawn from the study under any of the following circumstances: (1) the participant requests to discontinue treatment or withdraw from study participation; (2) a serious adverse event occurs that, in the judgment of the investigators or treating clinician, warrants discontinuation; (3) the treating clinician determines that modification of the treatment plan is clinically indicated because of a change in the participant’s condition; or (4) the participant is unwilling or unable to comply with the treatment protocol or study procedures.

## Outcomes

### Primary outcomes

The primary outcomes of this pilot trial are feasibility, safety, and preliminary clinical efficacy.

Feasibility will be evaluated using three indicators: recruitment feasibility, intervention adherence, and retention at the 6-month follow-up. Recruitment feasibility will be defined as the number of participants successfully enrolled during the 2-year recruitment period. Intervention adherence will be defined as the proportion of participants who complete all 20 planned treatment sessions. Retention will be defined as the proportion of enrolled participants who complete the 6-month follow-up assessment.

Safety will be evaluated by recording the incidence of adverse events and serious adverse events throughout the treatment and follow-up periods. The type, severity, outcome, and relationship of each adverse event to the intervention will be documented.

Preliminary clinical efficacy will be evaluated using the CDRS-R. CDRS-R total score will be assessed at baseline, at the end of treatment (week 4), and at follow-up weeks 8, 16, and 24. Response will be defined as a reduction of at least 50% in the CDRS-R total score from baseline, and remission will be defined as a CDRS-R total score of 28 or lower [28]. Response and remission will be determined at post-baseline assessments, using the baseline CDRS-R total score as the reference.

### Secondary outcomes

Secondary outcomes will assess changes in related clinical domains, including depressive symptoms, anxiety symptoms, suicidality, sleep quality, global clinical status, rumination, and health-related quality of life.

Depressive symptoms will be assessed using the Beck Depression Inventory-II (BDI-II). Anxiety symptoms will be assessed using the Hamilton Anxiety Rating Scale (HAMA) and the Screen for Child Anxiety Related Emotional Disorders (SCARED) [29]. Suicidality will be assessed using the Columbia-Suicide Severity Rating Scale (C-SSRS) [30]. Sleep quality will be assessed using the Pittsburgh Sleep Quality Index (PSQI). Global clinical status will be assessed using the Clinical Global Impression-Severity (CGI-S) and the Clinical Global Impression-Improvement (CGI-I) [31]. Rumination will be assessed using the Ruminative Responses Scale (RRS), and health-related quality of life will be assessed using the Pediatric Quality of Life Inventory 4.0 (PedsQL 4.0).

For secondary outcomes, changes from baseline will be evaluated where applicable. CGI-I will be assessed at post-baseline visits to evaluate overall clinical improvement relative to baseline. Secondary outcomes will be assessed at baseline, during treatment, at the end of treatment, and during follow-up up to 6 months, as specified in the assessment schedule. The C-SSRS will also be administered throughout the study to support ongoing safety monitoring of suicidal ideation and suicidal behaviors.

### Exploratory outcomes

Exploratory outcomes will include MRI-derived functional connectivity, cognitive performance, and multimodal neurophysiological and behavioral measures. MRI-derived functional connectivity will be analyzed using seed-based or whole-brain approaches to examine modulation of depression-relevant circuits. Cognitive performance will be assessed using THINC-it [32]. Multimodal exploratory biomarkers will be assessed, including electroencephalography (EEG), eye tracking, functional near-infrared spectroscopy (fNIRS), facial expression features, and acoustic features.

These exploratory measures will be collected according to the assessment schedule at baseline, at the end of treatment, and during follow-up where applicable. They will be used to characterize potential cognitive, neurophysiological, behavioral, and affective changes associated with treatment and are not designated as primary or secondary outcomes. The schedule of outcome assessments is shown in Table 1.

**Table 1:** Study of efficacy outcome measures.

Outcomes	Measure	Time points
Primary Outcomes	Recruitment Feasibility	At 2 years
	Intervention adherence	At 4 weeks
	Retention Rate	At 24 weeks
	Adverse events and serious adverse events recorded	At 2, 4, 8, 16 and 24 weeks
	CDRS-R	Response at the end of treatment (Week 4) (defined as≥50% reduction in CDRS-R from baseline) or Remission at the end of treatment (Week 4) (defined as CDRS-R ≤ 28)
Secondary Outcomes	BDI-II	At 2, 4, 8, 16 and 24 weeks
	HAMA	At 2, 4, 8, 16 and 24 weeks
	SCARED	At 2, 4, 8, 16 and 24 weeks
	C-SSRS	At 2, 4, 8, 16 and 24 weeks
	PSQI	At 2, 4, 8, 16 and 24 weeks
	PedsQL 4.0	At 2, 4, 8, 16 and 24 weeks
	CGI-I	At 2, 4, 8, 16 and 24 weeks
	CGI-S	At 2, 4, 8, 16 and 24 weeks
	RRS	At 2, 4, 8, 16 and 24 weeks
Exploratory Outcomes	MRI	At 4, 8, 16 and 24 weeks
	THINC-It	At 4, 8, 16 and 24 weeks
	EEG	At 4, 8, 16 and 24 weeks
	fNIRS	At 4, 8, 16 and 24 weeks
	Eye tracking	At 4, 8, 16 and 24 weeks
	Facial expressions	At 4, 8, 16 and 24 weeks
	Acoustic features	At 4, 8, 16 and 24 weeks

CDRS-R, Children’s Depression Rating Scale-Revised; HAMA, Hamilton Anxiety Rating Scale; BDI-II, Beck Depression Inventory-II; SCARED, Screen for Child Anxiety Related Emotional Disorders; C-SSRS, Columbia-Suicide Severity Rating Scale; CGI-I, Clinical Global Impression-Improvement Scale; CGI-S, Clinical Global Impression-Severity Scale; RRS, Rumination on Responses Scale.

### Data collection and management

Data will be collected using standardized questionnaires, clinical assessments, neuroimaging measures, and multimodal physiological or behavioral assessments, as applicable. All study instruments and outcome measures will be selected on the basis of established reliability and validity. Completed questionnaires and assessment records will be stored on a secure cloud-based platform, and results of clinical examinations and imaging assessments will be uploaded as formal reports. Data entry will be performed by authorized study personnel using access-controlled accounts to ensure data security, confidentiality, and integrity. Quality control procedures will include double data entry, routine data checks, and periodic verification to ensure accuracy and completeness. All data management processes will be documented and conducted in accordance with applicable regulations and institutional policies governing clinical trial data handling.

### Safety considerations and adverse events

Internationally accepted definitions of adverse events (AEs) and serious adverse events (SAEs) will be applied. An AE will be defined as any unfavorable and unintended medical occurrence in a participant receiving transcranial magnetic stimulation, regardless of whether it is considered related to the intervention. An SAE will be defined as any event that results in death, is life-threatening, requires hospitalization or prolongation of hospitalization, results in persistent or significant disability or incapacity, or involves a congenital anomaly or birth defect. After each treatment session, participants will be systematically asked about any adverse reactions or other medical events experienced, and all AEs and SAEs will be documented and managed in accordance with the trial’s safety procedures.

### Statistical analyses

#### Analysis populations and general principles.

All randomized participants who receive at least one treatment session will be included in the primary analysis set. Safety analyses will include all participants who receive at least one session of active or sham stimulation. Baseline characteristics will be summarized descriptively by treatment group. Continuous variables will be presented as mean and standard deviation or median and interquartile range, as appropriate, and categorical variables will be presented as frequencies and percentages.

As this is a pilot trial, the statistical analyses will focus primarily on estimation rather than definitive hypothesis testing. Effect estimates and corresponding 95% confidence intervals will be reported where appropriate, and P values will be interpreted cautiously as supportive rather than confirmatory. No formal adjustment for multiplicity is planned, given the exploratory nature of the trial.

#### Analysis of primary outcomes.

Primary outcomes will be analyzed descriptively in accordance with the pilot nature of the trial. Feasibility outcomes will include recruitment feasibility, intervention adherence, and retention at the 6-month follow-up. Recruitment feasibility will be summarized as the number of participants enrolled during the recruitment period. Intervention adherence and retention will be summarized as frequencies and proportions, with corresponding 95% confidence intervals calculated where appropriate.

Safety outcomes will be analyzed in the safety analysis set, defined as all participants who receive at least one session of active or sham stimulation. The incidence of adverse events and serious adverse events will be summarized by treatment group as the number and proportion of participants experiencing at least one event. Event counts, severity, outcomes, and relationship to the intervention will also be summarized descriptively.

Preliminary clinical efficacy will be evaluated in terms of response and remission based on CDRS-R total scores. Response and remission rates will be summarized descriptively by treatment group at the end of treatment and at each follow-up assessment, with 95% confidence intervals. Exploratory between-group comparisons may be conducted using logistic regression models at individual post-baseline time points or generalized estimating equations for repeated binary outcomes across follow-up, as appropriate. Effect estimates will be presented as odds ratios with 95% confidence intervals.

#### Analysis of secondary outcomes.

Secondary outcomes, including BDI-II, HAMA, SCARED, C-SSRS, PSQI, PedsQL 4.0, CGI-S, CGI-I, and RRS, will be analyzed according to outcome type. Repeated continuous outcomes will primarily be analyzed using linear mixed-effects models, where appropriate, with treatment group, time, and treatment-by-time interaction included as fixed effects and the corresponding baseline value included as a covariate. CGI-I will be analyzed as a post-baseline global improvement rating. Ordinal or categorical outcomes will be analyzed using appropriate generalized linear models, generalized estimating equations, ordinal regression models, or non-parametric methods, depending on the distribution and scale properties of the data. Results will be summarized at each prespecified assessment time point together with effect estimates and 95% confidence intervals where appropriate.

#### Analysis of exploratory outcomes.

Exploratory outcomes, including MRI-derived functional connectivity, THINC-it performance, EEG measures, eye-tracking measures, fNIRS measures, facial expression features, and acoustic features, will be analyzed using descriptive and exploratory approaches. For continuous exploratory measures, changes from baseline and between-group differences over time will be estimated using mixed-effects models or other suitable multivariable methods, where appropriate, depending on the structure and dimensionality of the data. Neuroimaging analyses will use seed-based or whole-brain approaches to characterize changes in depression-relevant circuits. Because of the pilot nature of the study and the large number of exploratory measures, these analyses will be regarded as hypothesis-generating.

### Missing data and sensitivity analyses

Patterns of missing data will be examined descriptively. For repeated continuous outcomes, mixed-effects models will serve as the primary analytic approach under the assumption that data are missing at random. Sensitivity analyses will be conducted, where appropriate, using multiple imputation, complete-case analysis, and alternative assumptions regarding missing data to assess the robustness of the findings. Additional sensitivity analyses may also be performed to examine the potential influence of protocol deviations or extreme values.

Exploratory subgroup analyses may be conducted, if data permit, according to selected baseline characteristics such as age, sex, and baseline depression severity. Given the small sample size, these analyses will be interpreted cautiously and considered hypothesis-generating.

### Safety analysis

Safety outcomes will be summarized descriptively by treatment group, including the type, frequency, severity, and incidence of adverse events and serious adverse events. Between-group comparisons will be conducted using the chi-square test or Fisher’s exact test, as appropriate. Tolerability-related indicators, including treatment interruption, dose reduction, or discontinuation, will also be summarized descriptively.

## Ethics and dissemination

### Ethical considerations

This investigator-initiated study at the First Affiliated Hospital of Chongqing Medical University is a randomized, double-blind, three-arm, sham-controlled, parallel-group pilot clinical trial. All study procedures will be conducted in accordance with the ethical standards of the relevant national and institutional committees on human experimentation and with the Declaration of Helsinki (1975), as revised in 2013. The study protocol was approved by the Ethics Committee of the First Affiliated Hospital of Chongqing Medical University on 21 August 2025 (Ethical Approval No. 2025-533-01), and the trial was registered at ClinicalTrials.gov on 15 September 2025 (Registration No. NCT07185438). The trial protocol and statistical analysis plan are publicly available through the trial registration record. Participant recruitment commenced on 1 September 2025 and is expected to be completed on 31 December 2026. Any protocol modifications, including changes to study procedures or participant safety measures, will be documented as protocol amendments and communicated in a timely manner to the Ethics Committee and relevant investigators. All amendments will be reviewed and approved by the Ethics Committee before implementation to ensure ongoing compliance with ethical standards.

Written informed consent will be obtained before enrolment. Because all participants are adolescents, the principal investigator will ensure that written informed consent is obtained from participants’ legal guardians and that assent or consent from participants is obtained in accordance with applicable ethical and legal requirements. Before consent is obtained, participants and their guardians will receive a clear explanation of the study procedures, potential risks, and anticipated benefits, and they will have the opportunity to ask questions.

Participants will be reimbursed for study-related examination costs incurred during the trial, and all required study equipment will be provided at no cost. Upon completion of study participation, participants will receive compensation for transportation expenses related to trial visits. In the event of any trial-related adverse events, necessary medical expenses will be covered and additional compensation will be provided in accordance with institutional policies and applicable regulations.

All personal information relating to potential and enrolled participants will be collected, stored, and shared in strict compliance with applicable privacy and data protection regulations. Appropriate safeguards will be implemented to protect confidentiality throughout the study, including secure data storage and access controls that restrict identifiable information to authorized study personnel only.

### Monitoring

A Data Monitoring Committee (DMC) will be established and will comprise experts in clinical research, biostatistics, and relevant clinical specialties from the First Affiliated Hospital of Chongqing Medical University. The primary responsibilities of the DMC are to oversee participant safety, review trial conduct and progress, and monitor data quality and integrity. The committee will review study progress at regular intervals throughout the trial.

At prespecified time points, the Principal Investigator and the DMC will review accumulating trial data, with particular attention to safety outcomes, study feasibility, and preliminary clinical signals. In the event of serious adverse events, major safety concerns, or other circumstances that may affect the ethical or scientific conduct of the study, the Principal Investigator and the DMC may recommend modification, temporary suspension, or early termination of the trial. Access to interim data will be restricted to DMC members, the Principal Investigator, and designated study personnel with authorization to review such data.

In addition, routine monitoring will be conducted by the Ethics Committee of the First Affiliated Hospital of Chongqing Medical University to ensure adherence to the protocol, protection of participants’ rights and welfare, and compliance with applicable regulatory requirements. Monitoring activities may include on-site visits, source data verification, and ongoing review of safety-related information.

### Dissemination

All participants and their guardians, where appropriate, will be provided with a summary of the study results. Findings will be published in peer-reviewed journals and disseminated to academic, professional, and public audiences through conferences and other appropriate communication channels, including social media where suitable.

### Patient and public involvement

Patients or the public will not be involved in the design, or conduct, or reporting, or dissemination plans of our research.

## Discussion

This study is a randomized, double-blind, three-arm, sham-controlled, parallel-group pilot trial designed to evaluate the feasibility, safety, and preliminary clinical efficacy of rs-fcMRI-guided rTMS targeting a circuit-informed DLPFC-sgACC axis in adolescents with MDD [33,34]. By directly comparing rs-fcMRI-guided individualized targeting with conventional left DLPFC stimulation and sham stimulation, the trial aims to clarify whether this imaging-guided neuromodulation approach can be delivered reliably in adolescents while also generating early clinical signals.

Although rTMS is an established treatment for adult depression and is generally associated with good tolerability and mostly mild, transient adverse effects [35], evidence in adolescent MDD remains limited and inconsistent. Existing studies differ substantially in design, stimulation targets, treatment parameters, and participant characteristics, which may partly explain the heterogeneity of reported findings. Moreover, the practical feasibility of delivering repeated rTMS sessions together with MRI and multimodal assessments in adolescents has not been adequately characterized. These uncertainties support the need for a pilot trial that addresses both clinical promise and real-world implementation.

The rationale for rs-fcMRI-guided targeting is grounded in adult neuroimaging evidence suggesting that antidepressant response to rTMS may be greater when stimulation is delivered to left DLPFC sites showing the strongest negative functional connectivity with the sgACC, a key node within depression-related neural circuits [36]. Compared with conventional targeting methods, this approach may allow more precise modulation of fronto-limbic networks involved in mood regulation. Accordingly, the present study will explore whether individualized rs-fcMRI-guided stimulation of the DLPFC-sgACC circuit is associated with more favorable preliminary clinical signals than standard left DLPFC stimulation in adolescents with MDD. It will also examine whether symptom improvement is accompanied by modulation of DLPFC-sgACC connectivity and broader changes in depression-relevant networks.

The principal value of this pilot trial lies in its ability to inform the next stage of clinical development. Feasibility and safety outcomes, including recruitment feasibility, intervention adherence, retention at the 6-month follow-up, and adverse event monitoring, will help determine whether protocol modifications are needed before a larger trial is undertaken. Additional trial process indicators, such as assessment completion, exploratory multimodal data completeness, tolerability-related findings, and blinding integrity, will further inform the feasibility of implementing the protocol in future studies. At the same time, preliminary clinical and circuit-level findings may guide the selection of outcome measures, follow-up schedules, and biomarker acquisition procedures for subsequent trials. Although this study is not designed to establish efficacy definitively or to develop predictive models, it may provide an initial empirical basis for more personalized rTMS strategies in adolescent depression and support the future standardization of individualized neuromodulation approaches in youth populations.

## Supporting information

S1 File SPIRIT checklist. (DOCX)
